# The Medical Humanities Council: A Model for Medical Student-Led Advancement of the Health Humanities

**DOI:** 10.1177/23821205251408645

**Published:** 2025-12-15

**Authors:** Joshua Anil, Phoebe Cunningham, Amanda Swain, Horace M. DeLisser

**Affiliations:** 1 Academic Programs Office, Perelman School of Medicine, 14640 University of Pennsylvania, Philadelphia, Pennsylvania, USA

**Keywords:** Health humanities, medical humanities, arts and humanities

## Abstract

This paper describes the Medical Humanities Council (MHC), a student-led initiative to promote the health humanities at the Perelman School of Medicine (PSOM) at the University of Pennsylvania. Building on data-focused advocacy, peer benchmarking, and collaboration with curricular leaders, the MHC has become the cornerstone of a sustainable and flourishing health humanities infrastructure and programming targeted at medical students at PSOM. The MHC illustrates how student advocacy can advance the arts and humanities and serves as a model for student-led promotion of the health humanities, particularly when programing and/or institutional support are lacking.

## Introduction

In aiming to understand how aspects of the human condition interplay with health and medicine, disciplines from across the humanities, arts, and social sciences have come together to form what is now known as the health humanities.^1^ While our efforts have focused on the arts and humanities in undergraduate medical education (UME), we have chosen for this commentary to use the term “health humanities” to emphasize their value in the training and support of health professionals more broadly. The scope of the health (medical) humanities is broad, which allows students and health practitioners to tailor health humanities programming around domains that resonate with themselves and their patient populations. For some, wellness through reflective writing or artistic sessions may hold the most weight. At other institutions, health humanities as a tool to engage with identity, belonging, health equity and social justice may be more applicable. Emerging from the FRAHME (Fundamental Role of the Arts and Humanities in Medical Education) initiative of the Association of American Medical Colleges,^2^ the Prism Model provides a guide for instruction and learning objectives in the humanities.^3,4^ This model lays out four steps to develop arts and humanities curricula in medical settings. It specifically highlights approaching humanities incorporation through the domains of mastering skills, perspective taking, personal insight, and social advocacy.

Despite the consensus articulated through the FRAHME initiative, the integration of the arts and humanities into U.S. medical school curricula and the student experience ranges widely. At medical schools where health humanities content and experiences are limited and/or institutional support is lacking, student initiative, effort and energy have been the source of programing and curricular change.^5^ As such, the integration of the arts and humanities at many institutions as envisioned by FRAHME will likely be driven, at least in part, by student action. In this piece, we describe the establishment of the Medical Humanities Council (MHC) by students at the Perelman School of Medicine (PSOM) at the University of Pennsylvania as well as its impact to date, offering it as a model for student advocacy for the health humanities.

## The Establishment of the Medical Humanities Council

**Figure 1.**
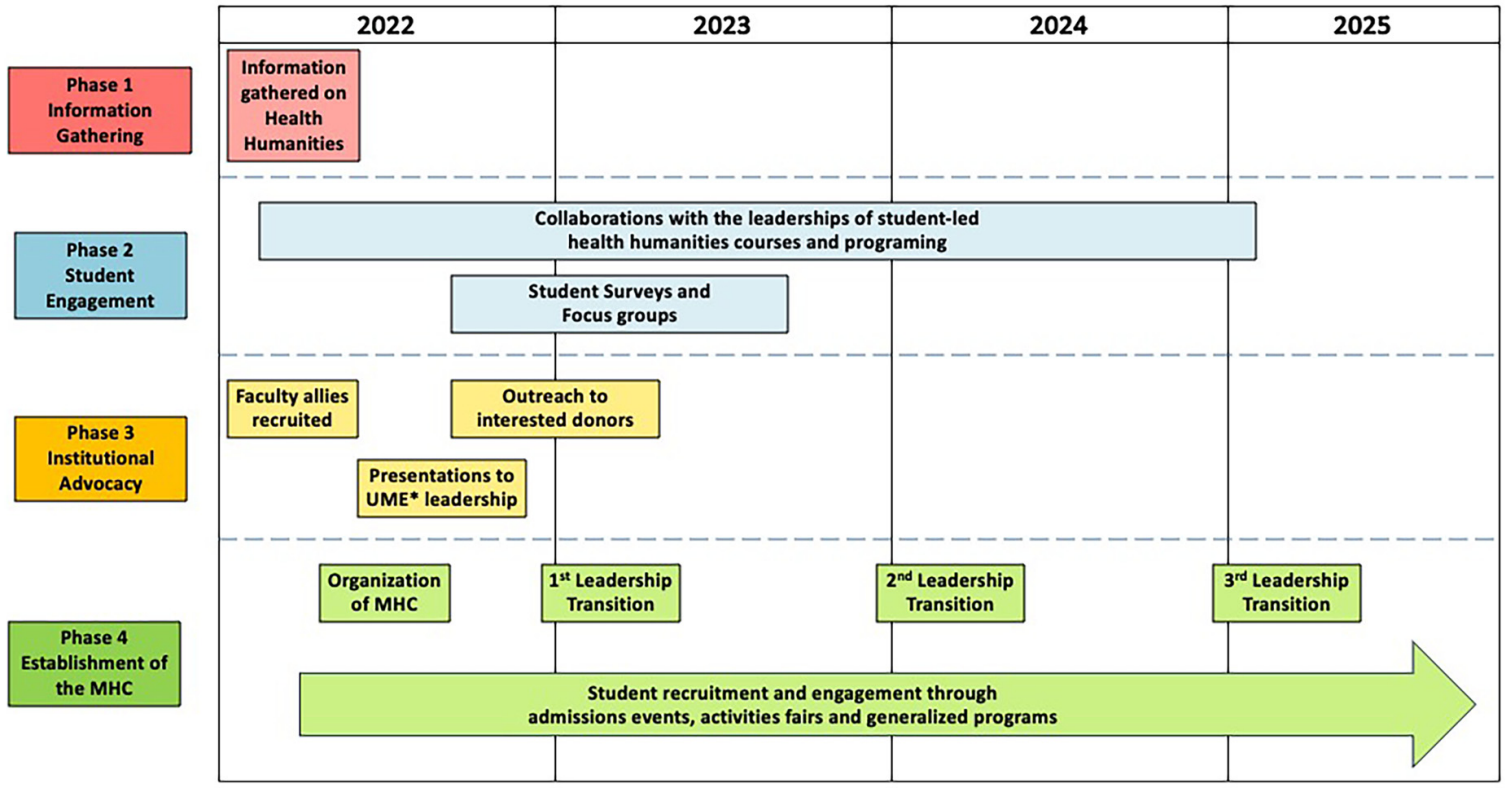
Timeline for milestone events in the development of the MHC. (*UME – undergraduate medical education).

Beginning with the recognition that there were significant opportunities for growth, two of the authors (JA, PC) took the lead in mobilizing their peers to enhance the health humanities programming and content at PSOM. Their leadership efforts, which are detailed below, involved four overlapping phases: gathering of information (Phase 1), student engagement (Phase 2), and institutional advocacy (Phase 3) that culminated in the creation of the MHC (Phase 4). This process is summarized in Figure 1, which provides a timeline of milestone events in the development and establishment of the MHC.

## Gathering Information on the Health Humanities at Peer Institutions

Initial efforts focused on characterizing the health humanities infrastructure at peer institutions to identify key components of health humanities programming that might be brought to PSOM (Figure 1, Phase 1). To do this systematically, we developed a scale to evaluate the health ecosystem of the arts and humanities at medical institutions, the Humanities and Arts Programming Score or HARPS, and used it to assess the health humanities programing at more than 30 medical schools, including our own.^3^ The evaluation focused on eight key areas: formal humanities infrastructure, faculty support, curricular integration, extracurricular engagement, longitudinal development opportunities, and research and scholarship. This process enabled us to identify our institutional strengths such as our robust extracurricular humanities student groups, as well as key areas for improvement and development, particularly our lack of an immersive humanities training track. Through this process we came to appreciate the diversity in structure and missions of medical humanities programing across the U.S. Additionally, while there were common elements we might seek to incorporate at our institution, ultimately the health humanities programing at PSOM would have to be one that aligned with our students’ specific interests and values.

### Understanding Student Perspectives on the Health Humanities

In parallel with the work to characterize the health humanities programing at peer institutions, efforts were also undertaken to understand the perspectives of PSOM students regarding the health humanities (Figure 1, Phase 2). This allowed us to gain information on how to develop programming that would engage a broad range of students with varying interests and/or experiences with the arts and humanities. Initial efforts involved meetings with the leadership of the health humanities student groups and organizations, soliciting their engagement in and support of collaborative efforts to promote the health humanities at PSOM, ultimately coalescing around the establishment of a medical humanities council. Additionally, student perspectives more broadly were captured using a survey tool we developed to assess current students’ (i) past experiences with and understanding of medical humanities (the term that was used in the surveys); (ii) current involvement with the medical humanities; (iii) barriers to participation in medical humanities-related activities; and (iv) interest in future medical humanity offerings. This survey was sent to all students and was complemented by focus groups aimed at gaining more qualitative perspectives.

### Advocacy for Institutional Support and Funding

The information gathered on the health humanities at peer institutions enabled the students to recruit faculty allies who facilitated access to and engagement of the leadership of undergraduate medical education (UME) at PSOM (Figure 1, Phase 3). Presentations to the UME leadership were compelling, particularly data highlighting where PSOM was lagging behind other institutions, resulting in additional commitments of institutional support and funding for the student-led health humanities initiatives. Presentation materials further included literature on the value of the humanities in physician development and well-being, important examples of arts and humanities programs across other institutions illustrative of how health humanities can meet student needs, the proposed MHC structure, and an implementation plan for the upcoming academic year. The presentation was also framed as an open discussion to get feedback on the MHC, the proposed programing, and the domains of an assessment of student perspectives. Important elements of this institutional support included enabling the students to work with PSOM's Office of Alumni Relations and Development to prepare promotional materials and to meet with interested donors to solicit funds to support student programming in the arts and humanities. These efforts have resulted in $3000 annually in donor support, supplemented by small amounts of money from our student government.

### The Structure of the Medical Humanities Council

**Figure 2.**
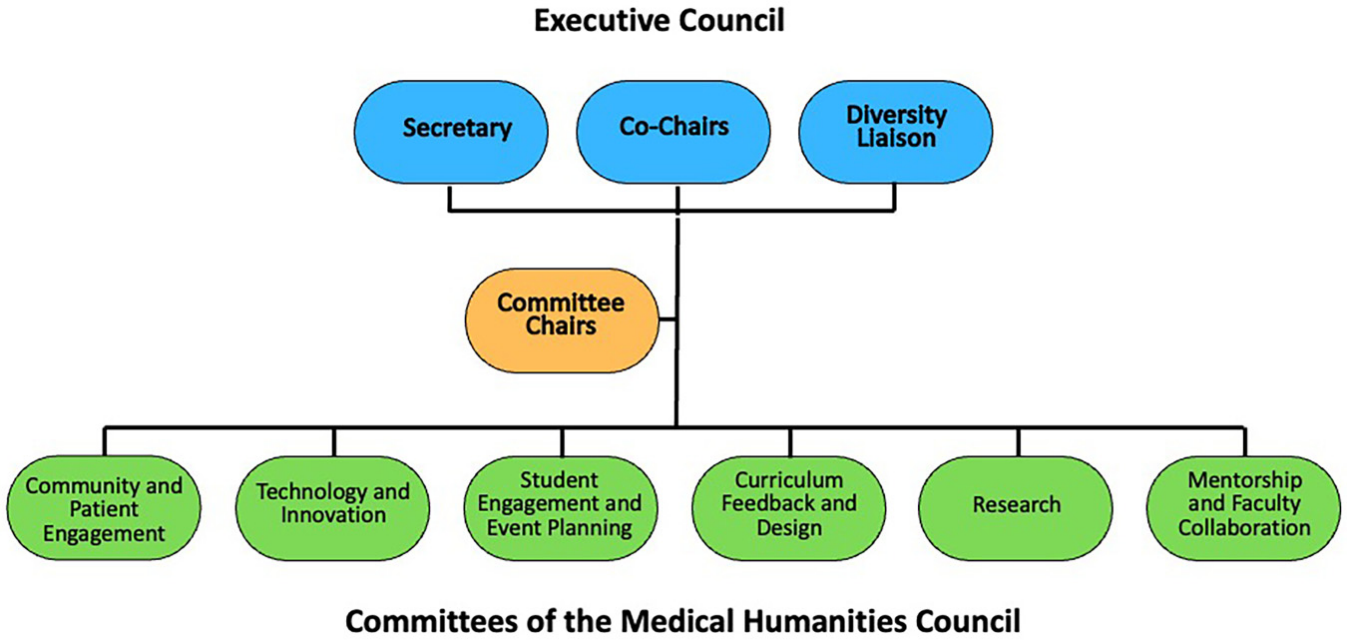
Initial organizational structure of the MHC.

With student and administrative support, the MHC was launched in 2022 (Figure 1 Phase 4). The initial MHC structure, depicted in Figure 2, was informed and then iterated by both our review of programming at other medical schools, as well as by the feedback from our student body and our faculty allies. Our discussions with students confirmed their eagerness for opportunities to plan programming and engage with their peers. Further, students at PSOM were particularly interested in the intersections of innovation and technology with the humanities. Additionally, our discussions with UME leadership and with our faculty allies reinforced the importance of being evidence-based and rigorously evaluating the impact and outcomes of our programing. Based on all these inputs, we established an organizational structure of six committees (community and patient engagement, technology and innovation, student engagement and event planning, curriculum feedback and engagement, mentorship and faculty collaboration, and research), with the committee chairs reporting to an executive leadership board comprised of two co-chairs, a secretary and a diversity chair (Figure 2). Over time this structure has evolved to better align with organizational needs and interests. The MHC has added a publicist, and treasurer, and consolidated its innovation and research committee.

## Initial Accomplishments, Outcomes and Impacts of the Medical Humanities Council

Over the past 4 years, the MHC has become the cornerstone of our medical school's health humanities infrastructure and programming, providing funding and an organizational home for the student projects, activities, initiatives related to the arts and humanities. Importantly, the MHC has signaled to students the value the school places on the health humanities as an important part of their medical education. The broad range of the activities and programming of the MHC are summarized in Table 1, but four major accomplishments are described in further detail below.

**Table 1.** Major Achievements of the MHC Over the Past Four Years.

Category	Achievements	Details
Sustainability	Successful leadership transitions	Three leadership transitions since the launch of the MHC in 2022 that have enabled continued active student engagement and programmatic growth
Curricular Enhancements	Humanities area of concentration	A robust for medical humanities track was established incorporating mentorship, scholarship, formal curricular training, and community engagement with transcript recognition.
	Iteration of the “Doctoring” course	Students worked with course directors to revise the existing humanities content in the mandatory pre-clerkship “Doctoring” course
	Student-initiated humanities-related courses	Elective opportunities in areas such as narrative medicine and drawing were developed by students
Research and Scholarship	Faculty mentorship database	A database was created of faculty profiles, organized by expertise and interests, to facilitate faculty-student engagement and professional development
	Accessible research database and tracker	A list was developed of active medical humanities research projects to facilitate student engagement with research opportunities
Student Programing	Support of humanities-focused student groups	Serving as a centralized home for student group leaders to find support, mentorship, additional funding, and cross-organizational resources
	Student coffeehouses and arts showcases	Biannual forums for student creativity via art, music, poetry, and writing
	Humanities focused lectures and presentations	Speaker selection of an annual lectureship in the health humanities and sponsorship of a health humanities lunch lecture series

### Institutionalization of the MHC

We cite the sustained student involvement in and commitment to the MHC over 4 years, with three very successful leadership transitions, as the MHC's most important accomplishment. Establishing an enduring institutional presence beyond the initial group of organizing students was identified as a primary strategic goal from the outset of this project during initial discussions with various stakeholder groups, and then students more broadly. This imperative drove the formation of an executive council and a committee structure composed of students drawn from all the medical school classes. Particular attention was given to recruiting first year students to serve on the fledgling MHC committees so that they might continue to participate in the MHC over their subsequent years of medical school. This recruitment begins before matriculation during “second-look” visits for accepted students, and then subsequently during the early months of the first year during activity fairs and through email messaging. The committee structure further provided flexibility in how intensively students could be involved and allowed them to focus on an area of the medical humanities that most resonated with their passions. The MHC has been especially fortunate to have successive co-chairs who have been passionate about the health humanities and dedicated to the MHC, with the committees serving as “the bench” from which the new MHC co-chairs have emerged. Additionally, quarterly check-ins with the two faculty advocates enabled their continued support.

### Establishment of an Area of Concentration in the Medical Humanities

During our primary survey of health humanities education, we became acutely aware that unlike some of our peers at other medical schools, our students did not have the opportunity to engage with the health humanities in a structured, longitudinal manner that provided for didactic and experiential learning coupled with consistent mentorship. To address this, the Medical Humanities Area of Concentration (AOC) was developed. While created independently and concurrently with the MHC, the administration of the AOC is now housed within the MHC under the purview of the Curricular committee. For three years, the MHC has led recruitment, handled administrative tracking of requirement completion, and developed programming and initiatives to help grow the concentration and make it a valuable opportunity to participating students. To date, over 80 students have participated in the concentration, and the first two sets of students have completed their capstone projects and graduated with this AOC and a formal transcript notation.

### Curriculum Iteration and Enhancement

The directors for our pre-clerkship Doctoring course in recent years had added art observation and reflection to the course's curriculum, but students often found these exercises frustrating and of little value. In response to these concerns, the course directors partnered with the MHC, which reviewed the existing lesson plans and then solicited feedback from students using an MHC-sponsored survey to help provide actionable feedback to the course directors. This collaborative effort culminated in a refresh of the arts and humanities content for the Doctoring course that has been well-received by students.

### Faculty Mentorship and Research Opportunities

An impetus for the creation of the MHC was the lack of a resource that students could access for opportunities, experiences and mentors pertinent to the arts and humanities. Our medical school resides within a large university near numerous humanities departments and scholars, with significant numbers of clinical faculty engaged in the health humanities throughout our affiliated health systems. Despite this wealth of people and resources in the health humanities space at our institution, students were frequently ignorant of what was available to them, and/or found it difficult to connect with those people or resources. The MHC therefore partnered with other student stakeholders to create a research opportunity list as well as a database of faculty from across the university and the associated health systems engaged in the arts and humanities that students could access to search for mentors. Subsequently, a formal pairing process was established to match students looking for mentors while also enabling faculty to recruit students directly for both creative and research projects. An additional benefit of this database has been to allow faculty from different areas of the University to become more aware of their colleagues’ work and interest, allowing for increased interdisciplinary collaboration.

## Implications and Future Directions

The energy and excitement for the MHC have grown well beyond what was envisioned when it was launched, becoming a student nexus for humanistic efforts at PSOM that did not exist just a few years ago. We believe this reflects an underlying desire for the health humanities as well as student recognition of the humanistic ethos that is at the core of medicine. In describing the development and initial success of the MHC we have sought to provide an approach and a framework that medical students might use to foster the health humanities at their school, particularly if such programming is absent and/or lacking institutional support. We also believe that MHC model might be applicable to other health professions trainees seeking to promote the health humanities at their institution. Our experience suggests that the elements for the success of an initiative of this type include a committed group of students, soliciting input and feedback from the general student body early in the process, identifying strong faculty advocates, and employing a data-driven approach to lobby for institutional recognition and support.

We, however, also acknowledge the challenges for others in attempting to replicate our MHC model. First, we recognize that for schools unaffiliated with a large university, or remote from the main university campus, it may be more difficult to access faculty, scholars and resources to support robust arts and humanities programing and experiences for students.^6^ Second, we benefited from an environment where there were already numerous examples of collaborative programming that brought humanities faculty into the clinical and medical education spaces.^7,8^ We, however, recognize that for other institutions, these types of programming or initiatives may be modest or absent, and so more effort would be required to launch an MHC-type initiative. Finally, the MHC has been fortunate, through the advocacy of its faculty advisors, to solicit and obtain donor funds of $3000 a year to support the operations of the organization. This model of funding may not be available to students at other medical schools. These issues therefore emphasize that with the arts and humanities being a broad enterprise, dependent on the context and their advocates, their integration into medical education will vary from one institution to another.

Additionally, as others have noted, the under-utilization of the arts and humanities in medical education represents a missed opportunity to enhance the training of medical students towards increased clinical and relational competence.^2,4^^9–18^ A number of studies have demonstrated the efficacy of health humanities interventions in providing objective improvement in reducing burnout and developing empathy, professionalism, tolerance of ambiguity, visual diagnostic skills, clinical judgement and interdisciplinary communication.^2,4^^15–18^ As a new, systems-level intervention, the MHC is currently working on developing more robust evaluation metrics to measure the outcomes of its programming. Initial efforts include a longitudinal study reassessing student engagement with the arts and humanities across each year of training, consisting of both extensive student surveys as well as semi-structured interviews to provide both qualitative and quantitative data. Additional methods for evaluation include continued qualitative assessment of the doctoring course via course evaluations, student comment pathways, and instructor feedback, and assessment of individualized elective outcomes, including visual analysis for an anatomical drawing course. Increasingly rigorous methods to assess key outcomes as advocated for by the FRAHME initiative^2–4^ and outlined in Eno 2023 are critical to support further humanistic integration,^19^ which we will look to employ as the MHC matures.

The success of the MHC provides a concrete demonstration of how student initiative and advocacy at an institutional level can push forward an agenda for bringing the arts and humanities into the medical student experience as proposed by the FRAHME initiative. While student action can be a catalyst for this integration, for the arts and humanities to assume its most impactful role in the education of medical students, institutional leadership ultimately needs to take the lead, even as the students continue their parallel efforts. Through comparisons to peer institutions and assessment of student interests in and desires for the health humanities, the gap between student led-initiatives and established institutional programs can be bridged. And as these types of student-driven, arts and humanities focused initiatives mature, their success in coalescing student interest, developing content and programing, and identifying the community of scholars and faculty engaged in the health humanities enables students to credibly push for a larger role of the health humanities in their medical education.
